# Does Dysphoria Lead to Divergent Mental Fatigue Effects on a Cognitive Task?

**DOI:** 10.1371/journal.pone.0130304

**Published:** 2015-06-15

**Authors:** Jesper F. Hopstaken, Sabine Wanmaker, Dimitri van der Linden, Arnold B. Bakker

**Affiliations:** 1 Department of Work and Organizational Psychology, Institute of Psychology, Erasmus University Rotterdam, the Netherlands; 2 Department of Clinical Psychology, Institute of Psychology, Erasmus University Rotterdam, the Netherlands; 3 King Abdulaziz University, Jeddah, Saudi Arabia; Istituto Superiore di Sanità, ITALY

## Abstract

**Objective:**

Tiredness, low energy, and listlessness are common symptoms to be associated with depression. The question remains to what extent these symptoms influence the effects of fatigue on sustained performance tasks, such as impaired task engagement and performance. Based on earlier findings, it was hypothesized that dysphoric (i.e., mildly depressed) individuals, compared to healthy controls, would display earlier fatigue onset and more severe fatigue effects on task engagement and performance during a cognitive task.

**Methods:**

Sixty-one dysphoric and twenty-one non-dysphoric control participants were compared during one hour of continuous performance on a 2-back task. During the task subjective fatigue, subjective engagement, objective task performance, baseline pupil diameter and stimulus-evoked pupil dilation were measured.

**Results:**

While we found that the dysphoric group reported relatively higher subjective fatigue than the healthy control group at the start of the experiment, we did not find any other divergent fatigue effects during the experimental task.

**Conclusion:**

One explanation for the absence of divergent effect is that dysphoria may not have such a profound impact on available cognitive resources, like attention, as initially thought. Based on the results of the present study, we conclude that dysphoria is not necessarily an increased risk factor for impaired sustained performance on cognitive tasks that may induce mental fatigue.

## Introduction

Depression has a high prevalence in the United States [1] and Europe [2] and negatively impacts work and academic performance [3,4]. This mood disorder is especially associated with difficulties in concentration [5], complex problem solving [5,6], and impaired work strategies [5]. Depression is generally known to negatively affect health [7,8], personal relationships [9], and productivity [10]. In the student population, depression is associated with lower educational performance [4,9,11] and higher drop-out rates from education [11]. Although in many cases depression leads to discontinued work activities, milder depressive symptoms (i.e., dysphoria) often go unnoticed within the working population [12]. The question remains to what extent these milder symptoms are associated with impaired work performance.

Second only to the depressed mood itself, tiredness, low energy, and listlessness are the most common symptoms to be associated with depression [13,14]. De Lecea, Carter and Adamantidis [15] have suggested that changes in arousal states and thresholds are at the core of most neuropsychiatric disorders, including depression which is correlated to structural hypo-arousal. Chaudhuri and Behan [16] describe that fatigue and cognitive disengagement in depression often result from a loss of interest and motivation. These authors also argue that even with usual levels of motivation, motor control, and sensory input, premature fatigue may arise because of unpleasant ambient conditions, dysautonomia, and underlying endocrine disturbances. Based on these findings, the question arises whether individuals with mild depressive symptoms (i.e. dysphoria) diverge from their non-depressive counterparts in their susceptibility to the effects of mental fatigue (i.e. earlier onset, more severe effects on performance). In this study, we will specifically address this question by comparing both groups while they are working on a cognitive task for an extended period of time.

Mental fatigue can occur as a chronic symptom of mental or physical disorders, but can also arise as an adaptive temporary condition resulting from prolonged cognitive effort (e.g., after a demanding workday). Mental fatigue is characterized by a reluctance for further effort and changes in mood motivation and information processing [17,18]. As a consequence, fatigued individuals have a tendency to disengage from the task at hand to prevent exhaustion. This disengagement is accompanied by lowered motivation (e.g. reduced effort), compromised cognition (e.g., diminished attention and task focus), and impaired performance [19,20]. Although these detrimental effects of fatigue have now been clearly established in healthy populations (e.g., students, healthy workers), there is a remarkable lack of studies testing how working on cognitively demanding and fatigue-inducing tasks affects individuals with subclinical depression. Yet, as it is known that individuals with dysphoric mood often also report chronic fatigue [14,16,21], it can be hypothesized that—compared to non-dysphoric individuals—they will be more susceptible to fatigue and disengagement while working on such tasks.

In the present study, we compare students with a dysphoric mood and healthy controls on task performance and subjective indices of mental fatigue. Moreover, we also examine the level of task engagement by measuring pupil dynamics. The diameter of the pupil has for many years been considered an index of psychophysiological arousal or neural gain, and the dilatory response has been linked to the occurrence of task-relevant events. Classic work of Beatty and Kahneman showed that the pupil is sensitive to momentary load and effort during mental tasks [22–24]. In recent years, this notion has evolved by specifically relating pupil diameter to task engagement and disengagement, based on cost-reward tradeoffs [25,26]. After working on a demanding task for a prolonged period of time, increasing levels of effort are required to maintain task focus. At some point, the effort no longer aligns with the potential benefits of engaging in the task. It has been argued and shown that the disengagement that results from this unfavorable tradeoff is reflected in decreased baseline pupil diameter and stimulus-evoked dilations toward task-relevant stimuli [27]. Based on the established association between dysphoric mood and heightened levels of chronic mental fatigue, we hypothesize that dysphoric individuals have lower average task engagement and display premature onset of mental fatigue effects on task engagement compared to healthy controls. More specifically,

Hypothesis 1: Individuals with dysphoric thoughts display higher average subjective fatigue and lower average subjective task engagement, baseline pupil diameter, stimulus-evoked pupil dilations and task performance compared to control individuals without dysphoric thoughts.

Hypothesis 2: Individuals with dysphoric thoughts display premature fatigue onset and effects reflected in earlier onset of increasing subjective fatigue and decreasing subjective task engagement, baseline pupil diameter, stimulus-evoked pupil dilations, and task performance compared to control individuals without dysphoric thoughts.

## Method

### Participants

Sixty-one dysphoric psychology students were compared to a control group of 21 non-dysphoric psychology students. The sample group for this study was part of a larger intervention study at a psychology institute of the university. Therefore, we were unable to perfectly balance the sample size of each of the groups. Although there is a noticeable difference in group size, modern analysis software use techniques that are generally robust to such an inequality of groups. The inclusion criterion was a Beck Depression Inventory–II (BDI-II) [28] score of minimally 10 for the dysphoric group and of maximally 5 for healthy group. We confirmed the statistically significant difference between the groups using an exact Mann-Whitney test for non-normally distributed data (U = 1239, z = 6.79, p < .01, r = .76, with a mean rank equal to 11 for the healthy control group and 51 for the dysphoric group). The mean BDI-II score of the dysphoric group was 20.2 (SD = 7.6, range: 10–46), which indicates an average depression [28] and 0.7 (SD = 1.1, range: 0–3) for the healthy group. The cut-off scores used for the selection are based on other studies comparing dysphoric and healthy students [29,30]. Information about age, gender, use of medication and medication and therapy history of both groups is reported in Table 1. All participants were well-rested and in good health as measured by self-reports. The participants reported to have slept seven or more hours and were asked to withhold the intake of caffeine and alcohol during the 24 hours before the experiment. All participants had normal or corrected to normal vision.

**Table 1:** Demographic Variables.

	Dysphoric group	Healthy control group
Gender (% men)	23.7	23.8
Age (*M*, *SD*)	20.8 (3.6)	21.4 (4.7)
% Current therapy	13.6	0.00
% History of therapy	35.6	0.00
% Use of medication for psychopathology	5.1	0.00

### Stimuli and data acquisition

Participants were seated in a dimly lit, and sound attenuated room facing an eye-tracking screen at a distance of approximately 65 cm. During the whole experiment, pupil diameter was measured continuously. The participants performed a visual letter 2-back task. The n-back task has been used successfully in previous experiments to induce fatigue [27,31]. It is a cognitively demanding task that requires the sustained engagement of working memory and attention in order to uphold adequate levels of performance [32].

### Procedure

The study was approved by the Medical Ethical Committee of the Erasmus University Rotterdam and registered at ClinicalTrials.gov (ID: NCT02184481). Students subscribed for the study through the psychology website and received credits for participation. All participants provided written informed consent to participate in the study. Participants practiced on the task until they reached a minimum of 70% accuracy. The experimental task lasted one hour and was divided into six time-on-task blocks. Each block consisted of 83 trails of the 2-back task and lasted for about 10 minutes (depending on random intervals). The n-back stimuli were displayed for 500ms with an inter-stimulus interval randomized at 5 to 5,5 seconds. The length of this interval was long enough to ensure that the pupil diameter returned to baseline levels [23,33].

### Measures and data processing

#### Subjective Measures

To measure time-on-task effects, the participants were asked “how tired do you feel?” after the practice trials and each time-on-task block during the experiment. They had to reply by moving a slider from 0 to 100, with increments of five. The slider had no anchors, but the extreme ends were labeled with “not at all” and “very much”. After answering this question we also asked “How engaged are you in the task?” before the task continued. Because these questions about subjective task engagement and fatigue were measured multiple times during the experiment, we could monitor the temporal progression with time-on-task. The participants had only limited time to answer the questions (i.e., ten seconds) to prevent them from resting between blocks.

#### Depression

We used the BDI-II [28] (Dutch version: [34]) to measure participants’ severity of depression symptoms. This self-report questionnaire contains 21 groups of statements about depression symptoms experienced the last two weeks. Adding up the scores of the questions, which range from 0 to 3, results in the total score. The reliability of this widely used questionnaire is good [35], with a Cronbach’s *α* of .94 in the present study.

#### Performance

The most relevant behavioral measure of performance on the n-back task is accuracy. We operationalized accuracy by calculating the d-prime for each time on task interval. As described by signal detection theory, the d-prime was calculated as an indication of accuracy [36]. While accuracy was the most important focus for the participant during the task, we wanted to make sure accuracy effects were not clouded by accuracy/speed tradeoffs. Therefore, we also examined reaction times (RTs).

#### Physiological measures

Pupil diameter was recorded continuously during the entire length of the experimental task with a Tobii Eyetracker 2150 with a sample rate of 50 Hz. The recordings were exported to Brain Vision Analyzer (Brain Products, Gilching, Germany). Artifacts and blinks were detected by the eye-tracker and removed by using a linear interpolation algorithm. To measure baseline pupil diameter, we averaged the pupil diameter in the 500ms before stimulus onset. During this period, the participants saw a fixation cross with the same luminosity as the letters, so there was no interference from eye reflexes to the environmental lightning. To analyze stimulus-evoked pupil dilation we performed a baseline correction for the 200ms before the stimulus onset. Then, we measured the positive peak within the first 1500ms after the onset of the stimulus. Trials in which performance errors occurred were excluded. The mean baseline pupil diameter and pupil dilation peak for each time-on-task interval were then exported to SPSS for further analysis.

### Statistical Analysis

The subjective, performance and pupil data were statistically analyzed using repeated measures ANOVA. Main and interaction effects of time-on-task and group (i.e. Dysphoric vs. Control) were tested over the six time-on-task blocks. To make sure that the effect of medication use did not influence the results and conclusions of the study, we did a parallel analysis that excluded the three participants that used medication. However, these parallel analyses showed that exclusion of these participants did not change the results in any meaningful way. Therefore, we only report the findings of the analyses that included these participants in the results section below.

## Results

### Subjective measures

**Fig 1:**
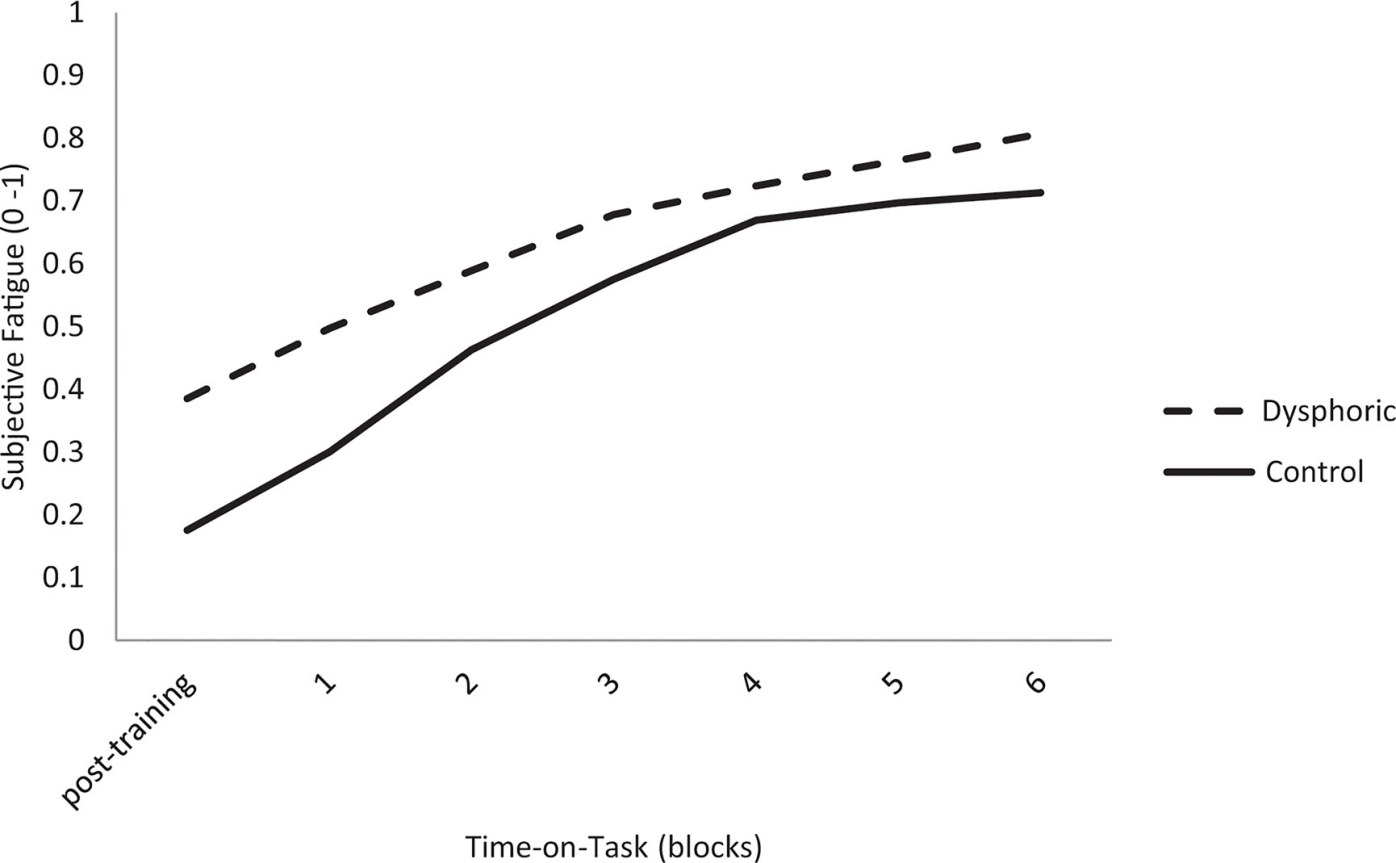
Interaction effect of time-on-task and group (dysphoric versus control) on subjective fatigue.

We analyzed the time-on-task effects for subjective fatigue and engagement during the experiment and found that fatigue significantly increased (*F*[6,384] = 109.4, *p* < .01, η_p_^2^ = .63) and engagement significantly decreased (*F*[6,384] = 10.8, *p* < .01, η_p_^2^ = .15) with increasing time-on-task. In contrast to our hypotheses, we did not find a significant group (dysphoric vs control; *F*[1,64] = 1.3, *p =* .25, η_p_^2^ = .02) and time-on-task x group interaction (*F*[6,384] = 1.4, *p* = .24, η_p_^2^ = .02) effect for engagement. We did however, find small group (*F*[1,64] = 4.1, *p* < .05, η_p_^2^ = .06) and interaction effect (*F*[6,384] = 3.1, *p* < .05, η_p_^2^ = .05) for subjective fatigue. Fig 1 shows that the dysphoric group scored higher on subjective fatigue—than the healthy control group—at the start of the experiment, but afterwards both groups scored similar. As can been seen in Table 2, which contains the means and standard deviations of all the observed measures during the experiment, the difference between the groups is especially large at the start of the experiment (dysphoric: M = .39, SD = .21; control: M = .18, SD = .20) which may explain these significant results. A follow up t-test, comparing the two groups, showed a significant difference in subjective fatigue during the first block of the experiment (t(64) = -2.89, *p* < .01), but not during the following blocks.

**Table 2:** Means and standard deviations of the observed variables.

	pre-training		block 1		block 2		block 3		block 4		block 5		block 6	
	D	C	D	C	D	C	D	C	D	C	D	C	D	C
Subjective fatigue	.39 (.21)	.18 (.20)	.50 (.25)	.30 (.21)	.59 (.24)	.46 (.25)	.68 (.23)	.58 (.28)	.72 (.24)	.67 (.27)	.76 (.23)	.70 (.29)	.81 (.19)	.71 (.32)
Subjective task engagement	.48 (.24)	.43 (.27)	.53 (.27)	.58 (.26)	.48 (.25)	.58 (.28)	.42 (.25)	.51 (.32)	.38 (.25)	.48 (.35)	.33 (.26)	.43 (.34)	.27 (.24)	.39 (.35)
d-prime	-	-	1.80 (1.21)	1.79 (1.23)	2.05 (1.08)	1.85 (1.80)	2.12 (1.00)	1.94 (1.77)	2.20 (1.17)	1.75 (1.54)	2.12 (1.14)	1.72 (1.85)	1.78 (1.21)	1.53 (1.75)
RT (ms)	-	-	1156 (353)	1039 (281)	1117 (354)	1003 (285)	1085 (324)	970 (294)	1072 (305)	996 (295)	1082 (331)	959 (277)	1035 (247)	978 (275)
Pupil diameter (mm)	-	-	5.19 (.86)	4.79 (.56)	5.06 (.80)	4.67 (.63)	4.97 (.75)	4.69 (.67)	4.93 (.78)	4.73 (.63)	4.94 (.80)	4.69 (.63)	4.94 (.77)	4.73 (.65)
Pupil dilation (mm)	-	-	.13 (.08)	.14 (.10)	.12 (.07)	.13 (.10)	.12 (.08)	.12 (.10)	.11 (.07)	.10 (.09)	.11 (.08)	.10 (.08)	.08 (.06)	.08 (.07)

Note: D = dyspohoric group, C = healthy control group.

### Behavioral measures

Analyzing the task performance data from block 1 through 6, we did not find a significant effect of time-on-task (*F*[5,320] = 1.9, *p* = .13, η_p_^2^ = .03). Looking at the mean performance at each block it became apparent that performance was relatively stable in the first part of the experiment and started to decline after the third block. The observation that task performance is stable or sometimes even increases during the start of the experiment is commonly found. This can be seen as a learning effect that can mask the effects of the onset of fatigue [37–39]. During the first two blocks of the experiment there was no change in d-prime (*F*[1,6] = 1.0, *p* = .31, η_p_^2^ = .02). However, from block three through six, d-prime significantly decreases (*F*[3,192] = 3.7, *p* = .01, η_p_^2^ = .06). RTs do not change significantly during the time of the experiment (*F*[5,320] = 2.4, *p* = .07, η_p_^2^ = .04) indicating there is no trade-off between speed and accuracy. While we found a main effect for time-on-task, we did not find a significant group effect (block 1 and 2: F[1,64] = 0.1, p = .74, ηp^2^ < .01; block 3 through 6: F[1,64] = 0.9, p = .36,ηp^2^ = .01) or time-on-task x group interaction effect for d-prime (block 1 and 2: F[1,64] = 0.4, p = .53, ηp^2^ = .01; block 3 through 6: F[3,192] = 0.5, p = .61,ηp^2^ = .01). We also did not find a significant group (F[1,64] = 1.5, p = .22,ηp^2^ = .02) and interaction (F[5,320] = 0.4, p = .77,ηp^2^ < .01) effect on RTs. These findings are not in line with our hypothesized differences between the dysphoric and control groups.

### Physiological measures

In line with previous studies, we found a decrease in both baseline pupil diameter (F[5,250] = 4.7, p < .01, ηp^2^ = .09) and peak pupil dilation (F[5,250] = 6.9, p < .01, ηp^2^ = .12) with increasing time-on-task. However, in contrast to our hypothesis and in line with most of the previously described analyses of this study, we did not find any significant group (baseline pupil diameter: F[1,64] = 1.7, p = .19, ηp^2^ = .03; Peak pupil dilation: F[1,64] < 0.01, p = .93, ηp^2^ < .01) or time-on-task x group interaction effects (baseline pupil diameter: F[5,250] = 2.2, p = .09, ηp^2^ = .04; Peak pupil dilation: F[5,250] = 0.6, p = .70, ηp^2^ = .01).

## Discussion

The present study examined whether individuals with dysphoric mood have higher average, and earlier onset of, negative effects of fatigue on task engagement and sustained performance during a cognitively demanding task. In line with previous findings of Hopstaken et al. [27], we found that subjective, performance, and psychophysiological measures of task engagement changed with increasing time-on-task. While we found that the dysphoric group reported relatively higher subjective fatigue than the healthy control group at the start of the experiment, we did not find any other divergent fatigue effects during the experimental task. This absence of divergent patterns contradicts our hypotheses, which we based on earlier work that showed that dysphoric mood is often associated with difficulties in concentration [5], complex problem solving [5,6], and impaired work strategies [5]. Therefore, it seems that a dysphoric mood does not necessarily increase the risk of more severe, or earlier, fatigue related decrements in engagement and performance on a cognitive task.

There are several explanations for the absence of divergent fatigue effects in dysphoric versus non-dysphoric students. One explanation is that dysphoria does not have such a profound impact on available cognitive resources, like attention, as initially thought. Although, some studies have suggested that the rumination that is associated with mood disorders uses cognitive resources that may diminish performance [40], others have observed that rumination often becomes a more automated bottom up process that has a relatively small load on cognitive resources [41,42]. In as far as the conclusions from the later studies are correct, it could be an explanation for why dysphoria does not have an additional detrimental effect on performance during mental fatigue. To follow up on this explanation, it could be interesting to see whether increasing or decreasing the cognitive load of the task (e.g., also include the 1-back and 3-back task) yields different results. Although previous studies have shown that specifically the 2-back task captures the cognitive effects of fatigue very well [27], it could be that dysphoria influences fatigue only in very low or very high cognitive load conditions. Another explanation for the absence of divergent fatigue effects may be found in sample we used. First of all, our groups consisted mainly of relatively young academic students. While evidence for an age-related explanation of dysphoria remains limited, there are studies suggesting that performance decrements are mainly visible in the elderly population [43]. Yet, there are other studies reporting the exact opposite [44].

Also with regard to the present sample, it must be noted that participants were dysphoric students, which indicates that they had a relatively low level of depression. Because few researchers have compared dysphoria and more severe depression directly, it is still an open question whether results obtained with dysphoric samples generalize to clinically depressed participants [1]. The use of a dysphoric, instead of a depression group, could also be seen as a strong feature of our study. The dysphoric group we used is much more likely to be part of the student or working population, while individuals with episodes of a depressive disorder are more likely to discontinue their study or work activities. Therefore, the impact of fatigue-related decrements in performance could potentially in practice have a much larger impact within the dysphoric population. Based on the results from our study however, we conclude that dysphoric mood is not necessarily an increased risk factor for impaired sustained performance on cognitive tasks that may induce mental fatigue.

## References

[1] Gotlib IH, Joormann J. Cognition and depression: current status and future directions. Annu Rev Clin Psychol. 2010;6: 285–312. 10.1146/annurev.clinpsy.121208.131305 20192795 PMC2845726

[2] Ayuso-Mateos JL, Vázquez-Barquero JL, Dowrick C, Lehtinen V, Dalgard OS, Casey P, et al Depressive disorders in Europe: prevalence figures from the ODIN study. Br J Psychiatry. 2001;179: 308–16. 10.1192/bjp.179.4.308 11581110

[3] Lerner D, Adler D a, Rogers WH, Chang H, Lapitsky L, McLaughlin T, et al Work performance of employees with depression: the impact of work stressors. Am J Health Promot. 2010;24: 205–13. 10.4278/ajhp.090313-QUAN-103 20073388 PMC4174367

[4] Hysenbegasi A, Hass SL, Rowland CR. The impact of depression on the academic productivity of university students. J Ment Health Policy Econ. 2005;8: 145–151. Available: http://thriveresearch.sidekickassociates.com/research-portal/articles/depression-college.pdf 16278502

[5] Lyubomirsky S, Kasri F, Zehm K. Dysphoric rumination impairs concentration on academic tasks. Cognit Ther Res. 2003;27: 309–330. 10.1023/A:1023918517378

[6] Lyubomirsky S, Nolen-Hoeksema S. Effects of self-focused rumination on negative thinking and interpersonal problem solving. J Pers Soc Psychol. 1995;69: 176–190. 10.1037//0022-3514.69.1.176 7643299

[7] Barth J, Schumacher M, Herrmann-Lingen C. Depression as a risk factor for mortality in patients with coronary heart disease: a meta-analysis. Psychosom Med. 2004;66: 802–13. 10.1097/01.psy.0000146332.53619.b2 15564343

[8] Knol MJ, Twisk JWR, Beekman a TF, Heine RJ, Snoek FJ, Pouwer F. Depression as a risk factor for the onset of type 2 diabetes mellitus. A meta-analysis. Diabetologia. 2006;49: 837–45. 10.1007/s00125-006-0159-x 16520921

[9] Fröjd S a, Nissinen ES, Pelkonen MUI, Marttunen MJ, Koivisto A-M, Kaltiala-Heino R. Depression and school performance in middle adolescent boys and girls. J Adolesc. 2008;31: 485–98. 10.1016/j.adolescence.2007.08.006 17949806

[10] Doris A, Ebmeier K, Shajahan P. Depressive illness. Lancet. 1999;354: 1369–75. 10.1016/S0140-6736(99)03121-9 10533878

[11] Szulecka TK, Springett NR, De Pauw KW. General health, psychiatric vulnerability and withdrawal from university in first-year undergraduates. Br J Guid Counc. 1987;15: 82–91. 10.1080/03069888708251646

[12] Fan ZJ, Bonauto DK, Foley MP, Anderson NJ, Yragui NL, Silverstein B a. Occupation and the prevalence of current depression and frequent mental distress, WA BRFSS 2006 and 2008. Am J Ind Med. 2012;55: 893–903. 10.1002/ajim.22094 22821712

[13] Stahl SM. The psychopharmacology of energy and fatigue. J Clin Psychiatry. 2002;63: 7–8. Available: http://europepmc.org/abstract/med/11838630 11838630 10.4088/jcp.v63n0102

[14] Demyttenaere K, De Fruyt J, Stahl SM. The many faces of fatigue in major depressive disorder. Int J Neuropsychopharmacol. 2005;8: 93–105. 10.1017/S1461145704004729 15482632

[15] De Lecea L, Carter ME, Adamantidis A. Shining light on wakefulness and arousal. Biol Psychiatry. Elsevier Inc.; 2012;71: 1046–52. 10.1016/j.biopsych.2012.01.032 22440618 PMC3771638

[16] Chaudhuri A, Behan P. Fatigue in neurological disorders. Lancet. 2004;363: 978–88. 10.1016/S0140-6736(04)15794-2 15043967

[17] Meijman TF. Mental fatigue and the efficiency of information processing in relation to work times. Int J Ind Ergon. 1997;20: 31–38. 10.1016/S0169-8141(96)00029-7

[18] Van der Linden D, Frese M, Meijman TF. Mental fatigue and the control of cognitive processes: effects on perseveration and planning. Acta Psychol (Amst). Elsevier; 2003;113: 45–65. 10.1016/S0001-6918(02)00150-6 12679043

[19] Boksem MAS, Tops M. Mental fatigue: costs and benefits. Brain Res Rev. Elsevier B.V.; 2008;59: 125–39. 10.1016/j.brainresrev.2008.07.001 18652844

[20] Hockey GR. Compensatory control in the regulation of human performance under stress and high workload; a cognitive-energetical framework. Biol Psychol. 1997;45: 73–93. Available: http://www.ncbi.nlm.nih.gov/pubmed/9083645 9083645 10.1016/s0301-0511(96)05223-4

[21] Bültmann U, Kant I, Kasl S V, Beurskens AJH., van den Brandt PA. Fatigue and psychological distress in the working population. J Psychosom Res. 2002;52: 445–452. 10.1016/S0022-3999(01)00228-8 12069868

[22] Kahneman D, Beatty J. Pupil Diameter and Load on Memory. Science (80-). American Assn for the Advancement of Science; 1966;154: 1583–1585. 10.1126/science.154.3756.1583 5924930

[23] Beatty J. Task-evoked pupillary responses, processing load, and the structure of processing resources. Psychol Bull. 1982;91: 276–92. Available: http://www.ncbi.nlm.nih.gov/pubmed/7071262 7071262

[24] Kahneman D. Attention and Effort [Internet]. The American Journal of Psychology. Englewood Cliffs, New Jersey: Prentice-Hall Inc.; 1973 Available: http://www.jstor.org/stable/1421603?origin=crossref

[25] Jepma M, Nieuwenhuis S. Pupil diameter predicts changes in the exploration-exploitation trade-off: evidence for the adaptive gain theory. J Cogn Neurosci. 2011;23: 1587–96. 10.1162/jocn.2010.21548 20666595

[26] Gilzenrat MS, Nieuwenhuis S, Jepma M, Cohen JD. Pupil diameter tracks changes in control state predicted by the adaptive gain theory of locus coeruleus function. Cogn Affect Behav Neurosci. 2010;10: 252–69. 10.3758/CABN.10.2.252 20498349 PMC3403821

[27] Hopstaken JF, van der Linden D, Bakker AB, Kompier MAJ. A multifaceted investigation of the link between mental fatigue and task disengagement. Psychophysiology. 2015;52: 305–15. 10.1111/psyp.12339 25263028

[28] Beck A, Steer R, Brown G. Beck Depression Inventory-II (BDI-II) San Antonio, TX: Psychological Corporation; 1996.

[29] Owens M, Derakshan N. The effects of dysphoria and rumination on cognitive flexibility and task selection. Acta Psychol (Amst). Elsevier B.V.; 2013;142: 323–331. 10.1016/j.actpsy.2013.01.008 23419810

[30] Owens M, Koster EHW, Derakshan N. Improving attention control in dysphoria through cognitive training: Transfer effects on working memory capacity and filtering efficiency. Psychophysiology. 2013;50: 297–307. 10.1111/psyp.12010 23350956

[31] Massar SAA, Wester AE, Volkerts ER, Kenemans JL. Manipulation specific effects of mental fatigue: evidence from novelty processing and simulated driving. Psychophysiology. 2010;47: 1119–26. 10.1111/j.1469-8986.2010.01028.x 20456663

[32] Watter S, Geffen GM, Geffen LB. The n-back as a dual-task: P300 morphology under divided attention. Psychophysiology. 2001;38: 998–1003. Available: http://www.ncbi.nlm.nih.gov/pubmed/12240676 12240676 10.1111/1469-8986.3860998

[33] Stern RM, Ray WJ, Quigley KS. Psychophysiological recording Oxford University Press, USA; 2000.

[34] Van der Does AJW. BDI-II-NL: Handleiding Beck Depression Inventory-II, Nederlandse vertaling en bewerking [BDI-II-NL: Manual Beck Depression Inventory-II, Dutch translation and adaptation] Lisse, The Netherlands: Swets Test Publisher; 2002.

[35] Evers A, Van Vliet-Mulder J, Groot C. Documentatie van tests en testresearch in Nederland, aanvulling 2005/01 (COTAN) Amsterdam, The Netherlands: Boom test uitgevers; 2005.

[36] Wickens TD. Elementary Signal Detection Theory [Internet]. Oxford University Press; 2001 10.1093/acprof:oso/9780195092509.001.0001

[37] Boksem M, Meijman T, Lorist M. Effects of mental fatigue on attention: an ERP study. Cogn brain Res. 2005;25: 107–16. 10.1016/j.cogbrainres.2005.04.011 15913965

[38] Lorist MM, Klein M, Nieuwenhuis S, De Jong R, Mulder G, Meijman TF. Mental fatigue and task control: planning and preparation. Psychophysiology. 2000;37: 614–25. Available: http://www.ncbi.nlm.nih.gov/pubmed/11037038 11037038

[39] Lorist MM, Boksem M a S, Ridderinkhof KR. Impaired cognitive control and reduced cingulate activity during mental fatigue. Brain Res Cogn Brain Res. 2005;24: 199–205. 10.1016/j.cogbrainres.2005.01.018 15993758

[40] Nolen-Hoeksema S. The role of rumination in depressive disorders and mixed anxiety/depressive symptoms. J Abnorm Psychol. 2000;109: 504–511. 10.1037/0021-843X.109.3.504 11016119

[41] Bargh J a., Tota ME. Context-dependent automatic processing in depression: Accessibility of negative constructs with regard to self but not others. J Pers Soc Psychol. 1988;54: 925–939. 10.1037//0022-3514.54.6.925 3397867

[42] Andersen SMS, Limpert C. Future-Event Schemas: Automaticity and Rumination in Major Depression. Cognit Ther Res. Kluwer Academic Publishers-Plenum Publishers; 2001;25: 311–333. 10.1023/A:1026447600924

[43] Hayslip B, Kennelly KJ, Maloy RM. Fatigue, depression, and cognitive performance among aged persons. Exp Aging Res. 1990;16: 111–5. 10.1080/07340669008251537 2090461

[44] Kim O, Kim A-J, Kim S-W, Baik S-H, Yang K-M. Fatigue, depression and sleep in young adult and middle-aged. Taehan Kanho Hakhoe Chi. 2003;33: 618–24. Available: http://www.ncbi.nlm.nih.gov/pubmed/15314414 15314414 10.4040/jkan.2003.33.5.618

